# Carotenoids Modulate FoxO‐Induced Cell Cycle Arrest in Human Cancer Cell Lines: A Scoping Review

**DOI:** 10.1002/fsn3.70100

**Published:** 2025-03-28

**Authors:** Zi Xin Lee, Hanting Guo, Aaron Deming Looi, Saatheeyavaane Bhuvanendran, Kasthuri Bai Magalingam, Wai Leng Lee, Ammu Kutty Radhakrishnan

**Affiliations:** ^1^ School of Science Monash University Malaysia Bandar Sunway Malaysia; ^2^ Food as Medicine Research Strength Jeffrey Cheah School of Medicine and Health Sciences, Monash University Malaysia Bandar Sunway Malaysia

**Keywords:** anticancer, cancer cell lines, carotenoids, cell cycle, FoxO

## Abstract

Carotenoids, a class of antioxidants, have shown great potential for cancer management. This scoping review aimed to elucidate the anticancer mechanisms of carotenoids by using a protein interactions and pathways approach. A literature search on five databases (Web of Science, PubMed, Ovid Medline, Ovid Embase and Scopus) was carried out, and studies investigating differential protein expression in cancer cell lines treated with carotenoids published in the last 10 years were included in the analysis. Sixty‐three research articles were short‐listed, and 17 carotenoids were used in these studies. The most studied carotenoids were fucoxanthin, astaxanthin, and crocin. The key cancer cell lines tested in these studies included breast, gastric, and lung cancers. Analysis of the proteins identified from these studies using the Search Tool for the Retrieval of Interacting Genes/Proteins (STRING) revealed the upregulation of proteins belonging to the pro‐apoptotic and FoxO signaling pathways. In contrast, several proteins in the PI3k/Akt and TNF signaling pathways and cell cycle regulation were downregulated, which can explain the observed anticancer effects. The findings from this scoping review suggest that the cell cycle arrest observed in carotenoid‐treated cancer cells may work through activation of the FoxO signaling pathway in these cells, highlighting their role as potential anticancer agents. Nonetheless, the lack of evidence on the pharmacology, pharmacokinetics, and physiology of carotenoids necessitates more robust and well‐designed clinical trials. Similarly, further investigations into the therapeutic effects of targeting the PI3K/Akt/FoxO axis to induce cell cycle arrest and its translational potential are required to ensure the successful development of effective treatments.

## Graphical abstract

Carotenoids upregulated pro‐apoptotic and FoxO signalling clusters. Carotenoids downregulated PI3k/Akt signalling, TNF signalling and cell cycle regulation clusters. Carotenoids may induce cell cycle arrest via the FoxO signalling pathway.

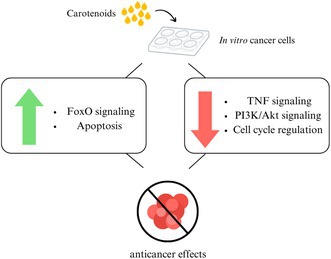

## 1 Introduction

Cancer is a group of more than 100 diseases that pose a significant public health challenge as it is the leading cause of premature death in 57 countries and one of the top three causes of premature death in about 120 countries (Bray et al. 2021). By the year 2024, it is estimated that there will be 28.4 million new cancer cases, which corresponds to a 47% increase compared to 2020 (Sung et al. 2021). It is projected that the most significant increase in cancer burden will be in countries with emerging human development index (HDI) levels due to the increased prevalence of cancer risk factors associated with higher HDI countries, such as unhealthy dietary patterns, smoking, and sedentary lifestyle (Sung et al. 2021). Although the current standards of care are cancer‐specific, the management of cancer generally involves surgery resection, radiotherapy, chemotherapy, or a combination of all. However, these methods have their limitations. For instance, radical surgery alone is ineffective due to the invasiveness of cancer cells at the microscopic level (Easaw et al. 2011; Gupta and Massagué 2006). Similarly, the use of radiotherapy poses the risk of radiotoxicity (Helissey et al. 2023; Mayer and Sminia 2008) while chemotherapy has resulted in the emergence of chemo‐resistant tumors (Yaghobi et al. 2021).

Carotenoids are hydrophobic pigments with an isoprenoid structure and a C40 backbone (Guest and Grant 2016). There are more than 600 natural carotenoids identified that can be broadly categorized into two groups, that is, carotenes and xanthophylls (Guest and Grant 2016). Carotenes refer to the parent hydrocarbon without oxygen atoms, while xanthophylls are their derivatives with oxygen atoms (Guest and Grant 2016). Xanthophylls are well‐known antioxidants, while carotenes are the precursors in the biosynthesis of vitamin A (Goodman et al. 1966). Vitamin A regulates oxidative balance, immune defense, and cell metabolism (Noh et al. 2019). As the human body cannot synthesize carotenoids (Goodman et al. 1966), these pro‐vitamin A carotenoids serve as an important dietary source for maintaining various biological functions. In recent years, there has been an increasing body of evidence that has reported the anticancer effects of carotenoids against several common cancer cell lines, including breast (Loganathan et al. 2015; Wang et al. 2022), gastric (Gao et al. 2019; Zhou et al. 2019), and colon (Cilla et al. 2015; Hormozi et al. 2019) cancers. Moreover, the lipophilic nature of carotenoids makes these compounds potential anticancer agents to target brain cancers as these compounds can penetrate the blood–brain barrier (BBB) (Craft et al. 2004; Yabuzaki 2017).

Despite the promising data, the safety risks associated with carotenoids remain concerning due to conflicting results obtained from clinical trials. For instance, two large‐scale clinical trials, namely, (i) the Alpha‐Tocopherol Beta‐Carotene Cancer Prevention (ATBC) trial and (ii) the Carotene and Retinol Efficacy (CARET) trial, were prematurely terminated due to evidence of increased cancer risks in the β‐carotene‐only arm (Group 1994; Omenn et al. 1996). However, conflicting results were observed when three other clinical trials showed no association between β‐carotene and incidences of cancer (Greenberg et al. 1990; Hennekens et al. 1996; Lee et al. 1999). Subsequent follow‐ups of the ATBC and CARET clinical trials concluded that while the observations could be attributed to several factors, it is likely that the higher serum β‐carotene concentration collected from the ATBC and CARET participants, 16‐fold and 12‐fold respectively, played a role in increasing the incidence of cancer (Goodman et al. 2004; Virtamo et al. 2003). In stark contrast, the Physicians' Health study, the Women's Health Study, and the Skin Cancer Prevention Study observed a lower serum β‐carotene concentration (Greenberg et al. 1996; Lee et al. 1999; Satterfield et al. 1990).

Interestingly, the pro‐cancer effects might be attributed to high doses of synthetic β‐carotene as natural β‐carotene, regardless of dosage, did not induce genotoxicity in pre‐clinical models (Grenfell‐Lee et al. 2014; Xue et al. 1998). Moreover, high doses of synthetic β‐carotene exerted pro‐oxidant activities under a free radicals‐rich environment (Duffield‐Lillico and Begg 2004; Palozza et al. 1995), which may be a plausible explanation for the results obtained in the ATBC and CARET clinical trials as the participants of both trials were either smokers or asbestos‐exposed workers, indicating their biological systems might have been under high oxidative stress for prolonged periods. In contrast, the three other trials (the Physicians' Health study, the Women's health Study and the skin Cancer Preventation Study) involved participants who were smokers and non‐smokers (Greenberg et al. 1990; Hennekens et al. 1996; Lee et al. 1999). Nonetheless, it remains elusive whether carotene and its origin play a role in cancer progression.

In the era of multi‐omics data, studying the cancer proteome can provide unique insights into the tumorigenesis of cancer through protein–protein interactions (PPI), post‐translational modifications, and peptide isoforms (Pandey and Mann 2000). Intrinsically, proteins are involved in the cellular structure and execution of biological activities. Thus, the identification of differentially expressed proteins is of spatial, functional, and temporal significance, as alterations in the cancer proteome are closely associated with tumorigenesis (Sallam 2015). Together with the integration of bioinformatic tools, proteomic studies of natural products may unravel druggable targets in cancers, elucidate the anti‐cancer mechanisms of natural products, and further contribute to their profiling for other potential uses (Muroi and Osada 2021). However, despite the benefits of the proteomics approach and the anti‐cancer potential of carotenoids, a comprehensive review of the anti‐cancer mechanisms of carotenoids remains unavailable. Thus, this scoping review aims to identify differentially expressed proteins in human cancer cell lines treated with carotenoids to elucidate their mechanisms of action.

## 2 Research Approach

The methodology used in this scoping review adhered to the five‐step framework outlined by Arksey and O'Malley (2005). Briefly, it involves the development of search strategies, followed by abstract and full‐text screening, data extraction, and finally, bioinformatics analysis.

### 2.1 Search Strategies

The development of search strategies was conducted by filling in a PICO worksheet template adapted from Miller and Forrest (2001). In this study, the search string used was “carotenes OR carotenoids OR α‐carotene OR β‐carotene OR lycopene OR lutein OR zeaxanthin” AND “anti‐cancer OR anti‐proliferative OR anti‐tumour OR anti‐apoptotic”. The search sources were restricted to five databases (Web of Science, PubMed, Ovid Medline, Ovid Embase and Scopus) published in the English language over the last 10 years. The inclusion and exclusion criteria of this study are provided in Table 1. While review studies, animal models and clinical trials may provide comprehensive information, they were excluded due to the lack of a consistent methodological framework such as the varying sample sizes, different target populations and distinct statistical approaches used to reach a conclusion. On the other hand, patient tissue cultures and case reports were also omitted as the results of these studies are often drawn from single‐patient data, which, if included, could introduce significant bias to our study. Thus, these studies were excluded to ensure meaningful data could be pooled for analysis.

**TABLE 1:** The inclusion and exclusion criteria used in the scoping review.

Inclusion criteria	Exclusion criteria
Human cancer cell lines	Animal models
Original research articles	Patient tissue cultures
Natural or synthetic carotenes	Reviews or meta‐analyses
Combination treatments with carotenes with carotene‐only data available	Clinical trials or practice guidelines Case reports
	Drug testing without carotenes

### 2.2 Study Selection and Data Extraction

All retrieved articles were independently screened by two independent reviewers. Duplicate articles and studies that did not fit the criteria were removed after each screening process. Any conflicts that arose throughout the process were resolved by a third independent researcher. Once the screening was completed, the shortlisted studies were subjected to data extraction using a data charting template created in Microsoft Excel. The charting process was conducted by two independent researchers, and any conflicts that arose during the process were resolved by a third independent researcher. Among the key information recorded were the type of cancer cell line used, type of intervention, names of proteins investigated, and the changes in their expression levels. The proteins were further classified based on their expression levels and reproducibility. Reproducible proteins were defined as proteins mentioned in more than one study.

### 2.3 Pre‐Processing of Protein Data

Due to the variations in protein naming across literature, all extracted protein data were first converted into gene symbols using the proteomic database UniProt (https://www.uniprot.org) (Consortium 2022) to streamline the analysis.

### 2.4 Identification of Reproducible and Differentially Expressed Proteins

To identify the reproducible and differentially expressed proteins (RDEPs), a Venn diagram was generated by inputting lists of reproducible proteins and differentially expressed proteins separately into the Draw Venn Diagram website (http://bioinformatics.psb.ugent.be/webtools/Venn/). The RDEPs were then defined based on the overlaps between groups. All non‐reproducible but differentially expressed proteins, as well as reproducible but non‐differentially expressed proteins, were excluded from subsequent analysis.

### 2.5 Protein–Protein Interaction Network Analysis

The protein–protein interactions (PPI) of reproducible and up‐regulated (RUPs) and downregulated (RDPs) proteins were analyzed by inputting the gene symbols of proteins into the multiple protein analysis mode in the Search Tool for the Retrieval of Interacting Genes/Proteins (STRING) (http://string‐db.org) database (Szklarczyk et al. 2015). The list of genes generated was screened to ensure an accurate input prior to mapping. All active interaction sources were selected, with the confidence score set to the highest (0.9) while *homo sapiens* was selected as the reference organism. The k‐means clustering option with a k value of 2 and 3 was used for analyzing the RUPs and RDPs, respectively. The pathways were identified via the analysis tools in STRING and validated using the Kyoto Encyclopaedia of Genes and Genomes (KEGG) Pathway Mapper (https://www.genome.jp/kegg/mapper/color.html).

## 3 Results

### 3.1 Search Results

**FIGURE 1:**
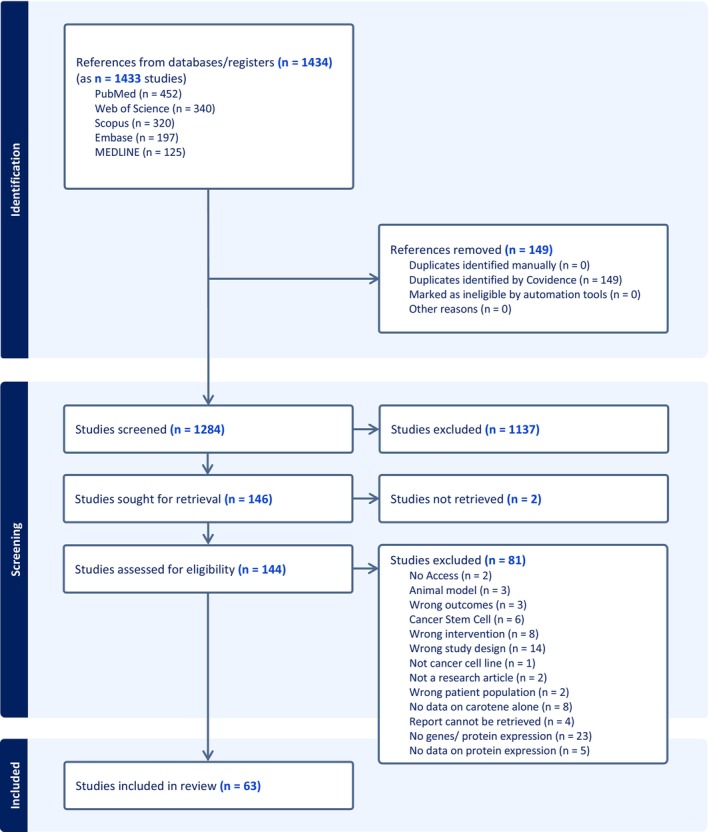
The Preferred Reporting Items for Systematic Reviews and Meta‐Analyses (PRISMA) flow chart (Page et al. 2021) outlining the study selection process in this scoping review. [n: Number].

The results from the literature search were summarized using the Preferred Reporting Items for Systematic Reviews and Meta‐Analyses (PRISMA) flowchart (Figure 1) (Page et al. 2021). Of the 1434 references obtained from the first search, 140 duplicate papers were removed. Following this, the title and abstract of 1284 papers were screened based on the inclusion and exclusion criteria (**Table** **2**), which resulted in 1137 studies being excluded from this study (Figure 1). Finally, two studies were excluded in the data retrieval step, and another 81 studies were removed during the full‐text screening step shown in the PRISMA chart, leaving 63 eligible studies, which were used for the data charting step (Figure 1).

**TABLE 2:** Summary of included studies (*n* = 63) and the differential protein expressions in various cancer cell lines.

References	Type of cancer (cell line)	Type of carotenoid	Proteins involved	Expression level
Bakshi et al. (2016)	MCF‐7 (breast cancer cells)	crocin	BAX, caspase‐3, caspase‐8, caspase‐9 Bcl‐2	up
				down
(Bakshi et al. 2020)	**BXPC3, Capan‐2** (pancreatic cancer cells)	crocin	BAX, caspase‐3, caspase‐9, cytosolic cytochrome c, p53, p38, p21cip1, P27kip1 BCL‐2, CDK2, c‐Myc	**up**
				**down**
(Bi et al. 2021)	**A431, SCL‐1** (cutaneous squamous cell carcinoma)	crocin	Cyclin D1 LC3 puncta, BECN1, LC3B, cleaved caspase‐3, ATG2B	**down**
				**up**
(Choi et al. 2014)	**HepG2** (human hepatoma cells) **PC‐3** (prostate cancer cells) **HT‐29** (colon cancer cells)	deinoxanthin	pro‐caspase‐3, BCL2 BAX	**down**
				**up**
(Cilla et al. 2015)	**Caco‐2** (colon cancer cells)	β‐cryptoxanthin + phytosterols (Betasitosterol, campesterol and stigmasterol)	cleaved‐PARP1 p‐Bad	**up**
				**down**
(Dong et al. 2013)	**SGC‐790** (gastric carcinoma cells)	β‐ionone	Cdk4, Cyclin B1, Cyclin D1, p‐ERK1/2 p27, p‐p38, p‐JNK	**down**
				**up**
(Du et al. 2015)	**LNCaP and PC‐3** (prostate cancer cells)	torularhodin	Bax Bcl2, androgen receptor (AR), prostate‐specific antigen (PSA)	**up**
				**down**
(Du et al. 2017)	**LNCaP, PC‐3** (prostate cancer cells)	torulene	Bax Bcl2, androgen receptor (AR), prostate‐specific antigen (PSA)	**up**
				**down**
(Erden 2020)	**MCF‐7** (breast cancer cells)	capsanthin	Bax, p53 Bcl2	**up**
				**down**
(Fang et al. 2022)	**NCI‐H1299, A549** (Non‐small cell lung cancer cells)	fucoxanthin	PCNA, cyclin D1, Bcl‐2, N‐cadherin, vimentin, phosphorylated PI3K, phosphorylated AKT Bax, cleaved‐caspase‐3, cleaved caspase‐9, E‐cadherin	**down**
				**up**

(Gansukh, Mya, et al. 2019)	**HeLa** (cervical carcinoma cells)	lutein	cleaved caspase‐3	up
(Gansukh, Nile, et al. 2019)	**HeLa** (cervical carcinoma cells)	beta‐cryptoxanthin	cleaved caspase‐3	**up**
(Gao et al. 2019)	**SGC‐7901, AGS** (gastric cancer cells)	beta‐cryptoxanthin	MMP‐2, MMP‐9, Cyclin E, Cyclin D1, CDK4, CDK6, PKA, eEF2K, AMPK p53, p21, cleaved caspase‐3, cleaved caspase‐8, cleaved caspase‐9, cyt C, p‐AMPK	**down**
				**up**
(Gong et al. 2018)	**MCF‐7 and MDA‐MB‐468** (breast cancer cells)	lutein	Bcl‐2 Bax, Hsp60, p‐p53	**down**
				**up**
(Hormozi et al. 2019)	**LS‐180** (colorectal cancer cells)	astaxanthin	superoxide dismutase, glutathione peroxidase	**up**
(Jeong et al. 2019)	**PANC‐1** (pancreatic cancer cells)	lycopene	cleaved caspase‐3, Bax Bcl‐2, p‐IĸBα, cIAP1, cIAP2, survivin	**up**
				**down**
(Karimian et al. 2022)	**MDA‐MB‐231, T47D** (breast cancer cells)	astaxanthin and melatonin	Bcl‐2	**down**
(Kavalappa et al. 2019)	**HepG2** (hepatocellular carcinoma cells)	beta‐carotene	caspase‐3, Bax Bcl‐2, phosphorylated Bad, PARP, NF‐kB (p105), NF‐kB (p55), Akt, ERK1/2, nuclear factor E2‐related factor 2 (Nrf‐2), superoxide dismutase‐2 (SOD‐2), HO‐1	**Up**
				**down**
(Kim et al. 2017)	**U266** (multiple myeloma cells)	crocin	p‐STAT, p‐JAK1, p‐JAK2, p‐Src, Bcl‐2, CXCR4, VEGF, Cyclin D1 SHP‐1, Bax, cleaved caspase‐3, cleaved caspase‐9	**down**
				**up**
(Kim and Park 2018)	**Hep3B, HepG2** (liver cancer cells)	crocin	p‐STAT3, p‐JAK1, p‐JAK2, p‐Src, Cyclin D1 CXCR4, VEGF, survivin, Bcl‐2 SHP‐1, Bax, cleaved caspase‐3, cleaved caspase‐9, cleaved PARP	**down**
				**up**
(Kim et al. 2020)	**SKBR3** (breast cancer cells)	astaxanthin	mutp53, SOD1, Pontin, Bcl2 cleaved PARP‐, Bax, cleaved caspase‐3, cleaved caspase‐9, pERK1/2, p‐JNK, p‐p38, SOD2	**down**
				**up**

(Kim et al. 2021)	**AGS** (gastric cancer cells)	astaxanthin	p‐RIP 1, p‐RIP 3, p‐MLKL	up
(Ko et al. 2016)	**A549, H1703** (non‐small lung cancer cells)	astaxanthin and Mitomycin C	Rad51, p‐AKT(Ser473)	**down**
(Lee et al. 2020)	**U937** (leukemia cells)	beta carotene + interleukin 4 (IL‐4)	CD163, (peroxisome proliferator = −activated receptor gamma (PPARγ)	**down**
	**HCT116** (colon cancer cells)	beta carotene + M2 macrophages‐conditioned media (CM)	CD133, CD44, Sox2	**down**
		beta carotene + TGFβ‐1 beta‐carotene + activated fibroblasts CM	α‐SMA, vimentin E‐cadherin	**down**
				**up**
(Li et al. 2015)	**LM3, SMMC**‐**7721** (hepatocellular carcinoma cells)	astaxanthin	proliferating cell nuclear antigen (PCNA), Bcl‐2, IKK‐β, NF‐κB p65 (nucleus), p‐NF‐κB p65, p‐IKKα/β, GSK‐3β, β‐catenin, pGSK‐3β, PI3K, p‐Akt, ERK, p‐ERK Bax, caspase‐3, caspase‐9, IκB‐α	**down**
				**up**
(Li et al. 2018)	**MDA‐MB‐157, MCF‐7** (breast cancer cells)	lutein	HES1, vimentin, N‐cadherin, HIF‐1α, NOTCH3, JAG1, JAG2, DLL1 E‐cadherin	**down**
				**up**
(Liao et al. 2016)	**H1650, H1703** (lung carcinoma cells)	astaxanthin and permetrexed	thymine synthase, p‐MKK 1/2, p‐ERK 1/2	**down**
(Liu et al. 2013)	**HepG2** (hepatoblastoma cells)	fucoxanthin and cisplatin	NF‐κB, p‐p38, p‐ERK, p‐PI3K/Akt p‐IκB‐α	**down**
				**up**
(Liu et al. 2016)	**U87, U251** (glioblastoma)	fucoxanthin	Bcl‐2, matrix metalloprotease 2 (MMP‐2), MMP‐9, urokinasetype plasminogen activator (uPA), p‐Akt, p‐mTOR, p‐p38 Bax, p‐PARP, p‐caspase‐9, p‐caspase‐3, H2AX, p‐ERK	**down**
				**up**
(Loganathan et al. 2015)	**MCF‐7, MDA‐MB‐231** (breast cancer)	total mixed carotenoids	cleaved PARP NF‐κB p65	**up**
				**down**
(Luan et al. 2022)	A549, NCI‐H1299 (lung adenocarcinoma)	fucoxanthin	Bax, cleaved caspase‐3, cleaved PARP, E‐cadherin Bcl‐2, caspase 3, PARP, vimentin, β‐catenin	up
				down

(Manmuan and Manmuan 2019)	**SW‐620** (colorectal cancer)	fucoxanthin and 5fluorouracil (5‐FU)	matrix metalloproteinase 9 (MMP‐9)	down
(Mei et al. 2017)	**A549, H1299** (non‐small cell lung cancer cells) **A549** only	fucoxanthin	p21^waf1/^cip1, Fas, PUMA, caspase‐3, caspase‐8 BCL‐2 p53, p‐p53	**up**
				**down**
				**up**
(Ming et al. 2021)	**A549, H1299** (non‐small cell lung cancer cells); **H446** (small cell lung cancer)	fucoxanthin	Snail, Twist, MMP‐2, Fibronectin, N‐cadherin, PI3K, p‐Akt, NFκB p65 Tissue inhibitors of metalloproteinase 2 (TIMP‐2)	**down**
				**up**
(Moccia et al. 2020)	**HG3** (chronic lymphocytic leukemia using lymphoblastoid cell derived from B1 cell immortalized with EBV infection)	total mixed carotenoids	LC3‐I, LC3‐II, p‐AMPK^Thr372^, p27^Kip1^ p62, AMPK	**up**
				**down**
(Oliveira et al. 2017)	**MCF‐7** (breast cancer cells); **Caco‐2** (colon cancer cells); **MKN‐28** (stomach cancer cells)	crocetin	IL‐1β	**down**
	**MCF‐7** (breast cancer cells)		TNF‐α IL‐6	**down**
				**up**
(Park et al. 2019)	**CRL 1739** ( *Helicobacter pylori* ‐induced gastric adenocarcinoma cells)	beta carotene	TRAF1, TRAF2, NADPH oxidase, NF‐κB (nuclear)	**down**
(Petchsak and Sripanidkulchai, 2015)	**MCF‐7** (breast cancer cells)	lycopene	Bax, caspase‐6, caspase‐8, caspase‐9	**up**
(Sawant et al. 2019)	**HeLa** (cervical carcinoma cells); **MDAMB‐231** (breast cancer cells)	crocin	α‐tubulin, EB1 bound to the polymeric fraction of tubulin acetylated tubulin, γ‐H2AX foci, BubR1, Mad2	**down**
				**up**
(Sheng et al. 2020)	**AGS, KATO‐3, MKN‐28, MKN‐45, NCI‐** **N87, YCC‐1, YCC‐6, YCC‐16, SUN‐5**, **SUN‐216, SUN‐484, SUN‐668** (gastric cancer cells)	zeaxanthin	IκBα Bcl‐2, pro cas‐3, pro‐PARP, p‐ERK, p‐STAT3, STAT3, NF‐κB, p‐ AKT, CDK1/2, Cyclin A, Cyclin B1	**up**
				**down**
(Siangcham et al. 2020)	**A172** (glioblastoma cells)	astaxanthin	MMP‐2, MMP‐9	**down**
	**MCF‐7** (breast cancer cells)	beta carotene	caspase‐3, cleaved caspase‐3, cleaved PARP	**up**
(Sowmya Shree et al. 2017)			Bcl‐2, p‐Bad, PARP, p‐ERK1/2, p‐Akt, NF‐κB p65 (nucleus), NFκB p65 (cytosol), Nrf2, SOD2, XBP‐1	**down**
(Su et al. 2019)	**SKOV3** (ovarian carcinoma cells)	astaxanthin and human serum albumin (HSA)	NF‐κB p65, p‐IκBα, IKKβ, p‐IKKα/β, Bcl‐2, Cyclin D1, c‐Myc, XIAP, p‐ERK1/2, ERK1/2 cytochrome c, caspase‐3, caspase‐9, Bax, p53, p‐p38, p38, γH2AX	**down**
				**up**
(Sun, Zhao et al. 2020)	**DU145** (prostate cancer cells)	astaxanthin	STAT3, JAK2, NF‐κB p65, Bcl‐2 caspase‐3, caspase‐9, Bax	**down**
				**up**
(Takeshima et al. 2014)	**MCF‐7, SK‐BR‐3, MDA‐MB‐468** (breast cancer cells) **MDA‐MB‐468** only	lycopene	ERK1/2, p‐ERK, p21, PARP, cleaved PARP Cyclin D1 Bax p‐Akt, Akt, p‐mTOR, mTOR	**up**
				**down**
				**up**
				**down**
(Tang et al. 2022)	**TPC‐1, IHH‐4** (papillary thyroid cancer cells)	crocin	caspase‐3, protein tyrosine phosphatase 4 (PTPN4)	**up**
(Terasaki et al. 2017)	**DLD‐1** (colorectal cancer cells)	fucoxanthinol	Cyclin D1, intergrin β1 NF‐κB p50, NF‐κB p52, PPARγ, p‐Akt^ser473^, p‐FAK^Tyr397^	**up**
				**down**
(Wang et al. 2014a)	**T24** (bladder cancer cells)	fucoxanthin	CDK‐2, CDK‐4, cyclin D1, cyclin E, mortalin p21, cleaved caspase‐3, p53	**down**
				**up**
(Wang et al. 2020)	**CAL‐27, SCC‐9** (oral cancer cells)	lycopene	Bcl‐2, p‐PI3K, Akt, p‐Akt, p‐mTOR, N‐cadherin Bax	**down**
				**up**
	**SCC‐9** only		E‐cadherin	**up**
(Wang et al. 2022)	**MCF‐7** (breast cancer cells)	fucoxanthin	Zeb1, Snail, Twist, N‐cadherin, β‐catenin, PI3K, p‐PI3K^Tyr458^, Akt 1/2/3, p‐Akt1/2/3 (AKT1‐Tyr 315/AKT2‐Tyr316/AKT3‐Tyr312), p‐FAK(Tyr397), FAK, p‐PTK2, p‐Paxillin^(Tyr118)^, Paxillin, p‐CFL1^(Ser3)^	**down**
(Wu et al. 2019)	**U251** (glioma cells)	fucoxanthin	caspase‐3, caspase‐8, caspase‐9, cleaved PARP, p‐ATM^Ser1981^, p‐ATRSer428, p‐p53Ser15, p‐histoneSer139, p‐JNKThr183, p‐38Thr180, pERKThr183 p‐Akt^Ser473^	**up**
				**down**
(Wu et al. 2021)	**MDA‐MB‐231** (breast cancer cells)	capsanthin	p21	**up**
			cyclin A, EZH2, H3K27me3	**down**
(Xu et al. 2019)	**SKOV3** (ovarian cancer cells)	lycopene	Bax Bcl‐2	**up**
				**down**
(Xu et al. 2022)	**MDA‐MB‐231, MDA‐MB‐468, BT‐549**, **MCF‐7** (breast cancer cells)	crocin	p65 p‐p65	**up**
				**down**
	**BT‐549** and **MCF‐9** only		TNF‐α, IL‐1β, PRKCQ	**down**
(Yamagata et al. 2017)	**A549** (lung cancer cells)	lutein	Bax Bcl	**up**
				**down**
(Ye et al. 2014)	**HeLa** (cervical cancer cells)	fucoxanthin	Bax Bcl‐2, p‐Akt, NF‐κB (nuclear)	**up**
				**down**
(Yu et al. 2018)	**SGC‐7901, BGC‐823** (gastric cancer cells)	fucoxanthin	Mcl‐1, STAT3, p‐STAT3	**down**
(Zeng et al. 2018)	**HepG2** (hepatocellular carcinoma)	fucoxanthin and fucoxanthinol	Bcl‐2 Bax	**up**
				**down**
(Zhang et al. 2016)	**Eca109** (esophageal squamous cell carcinoma)	Beta carotene and 5fluororacil (5‐FU)	Cav‐1, p‐AktSer475, p‐mTORSer2448, p‐p70s6kThr389, p‐NF‐κBSer536, Bcl‐2 Bax, caspase‐3	**down**
				**up**
(Zhang et al. 2021)	**FTC‐133** (thyroid cancer)	crocin	caspase‐8, caspase‐9 p‐JAK2, JAK2, p‐STAT3, STAT3, p‐ERK	**up**
				**down**
(Zhao et al. 2014a)	**K562** (chronic myeloid leukemia)	beta carotene/bixin/astaxanthin and rosiglitazone	PPARγ, p21 cyclin D1	**up**
				**down**
(Zhou et al. 2016)	**HGC‐27** (gastric cancer)	lycopene	LC3‐I, p‐ERK	**up**
(Zhou et al. 2019)	**HGC‐27, AGS** (gastric cancer cells)	crocin	KLF5, HIF‐1α, N‐cadherin, Snail E‐cadherin	**down**
				**up**

### 3.2 Study Characteristics

**FIGURE 2:**
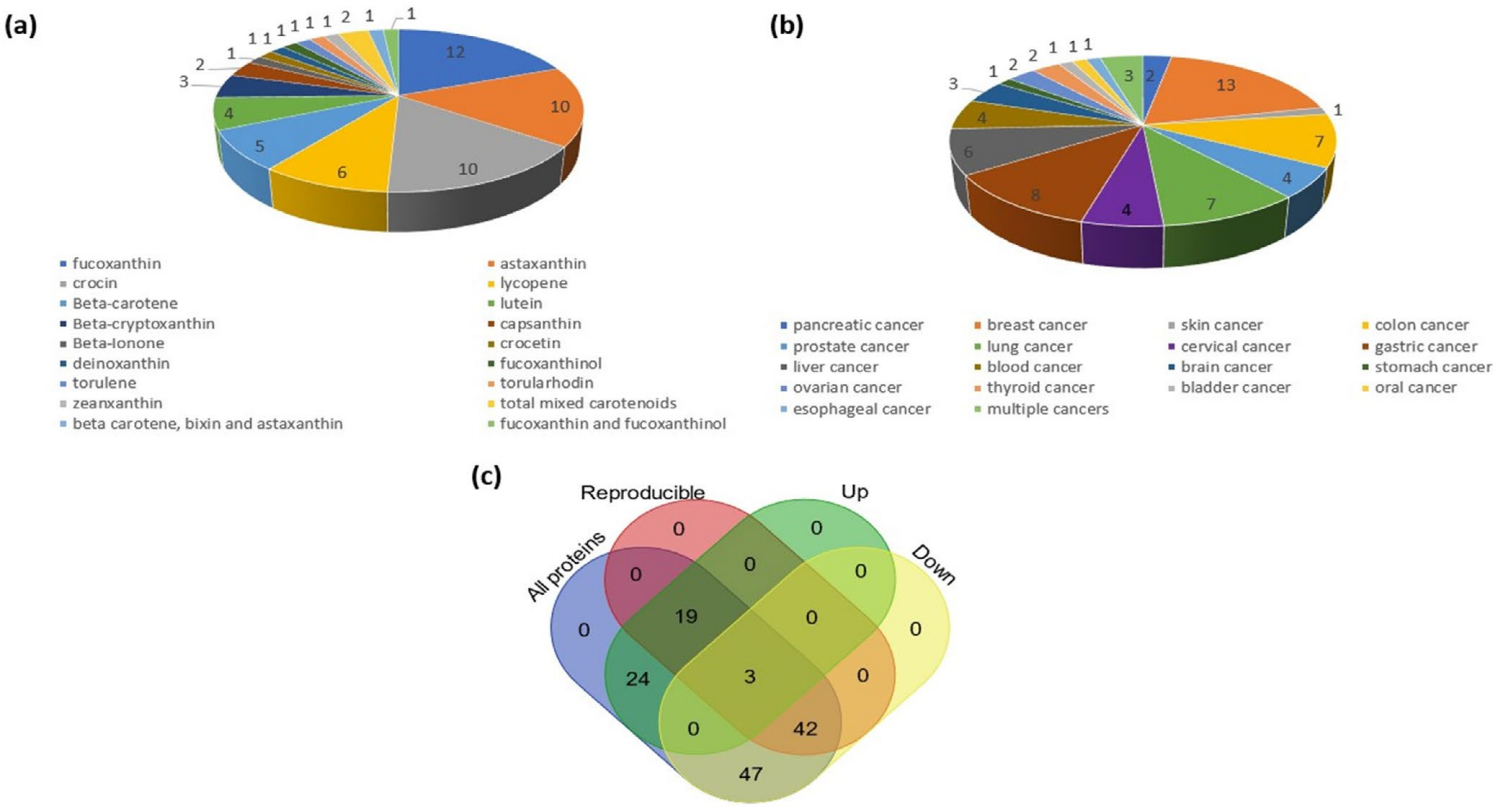
An overview of the study characteristics of the papers (*n* = 63) identified in this scoping review showing (a) the number of papers that used carotenoids as an intervention, (b) the number of papers that studied different types of cancer, and (c) Venn diagram illustrating the overlaps between all the up‐ and down‐regulated proteins identified in this scoping review. The list of proteins in each category can be found in the Appendix (Table S1).

The characteristics of the 63 studies selected for analysis in this scoping review are summarized in **Table** **2**. Briefly, 59 out of the 63 (59/;63) studies evaluated the anticancer effects of a single carotenoid. Only four (4/63) studies used different types of carotenoids as interventions (Figure 2a). Of the 59 studies, 12 studies used fucoxanthin as an intervention (Fang et al. 2022; Liu et al. 2013; Liu et al. 2016; Luan et al. 2022; Manmuan and Manmuan 2019; Mei et al. 2017; Ming et al. 2021; Wang, Zeng, et al. 2014a; Wang et al. 2022; Wu et al. 2019; Ye et al. 2014; Yu et al. 2018), 10 studies used astaxanthin (Hormozi et al. 2019; Karimian et al. 2022; Kim et al. 2020; Kim et al. 2021; Ko et al. 2016; Li et al. 2015; Liao et al. 2016; Siangcham et al. 2020; Su et al. 2019; Sun, Zhao, et al. 2020), 10 studies used crocin (Bakshi et al. 2016, 2020; Bi et al. 2021; Kim et al. 2017; Kim and Park 2018; Sawant et al. 2019; Tang et al. 2022; Xu et al. 2022; Zhang et al. 2021; Zhou et al. 2019), six studies used lycopene (Jeong et al. 2019; Petchsak and Sripanidkulchai 2015; Takeshima et al. 2014; Wang et al. 2020; Xu et al. 2019; Zhou et al. 2016), five studies used β‐carotene (Kavalappa et al. 2019; Lee et al. 2020; Park et al. 2019; Sowmya Sowmya Shree et al. 2017; Zhang et al. 2016), four studies used lutein (Gansukh, Mya, et al. 2019; Gong et al. 2018; Li et al. 2018; Yamagata et al. 2017), three studies used β‐cryptoxanthin (Cilla et al. 2015; Gansukh, Nile, et al. 2019; Gao et al. 2019), two studies used capsanthin (Erden 2020; Wu et al. 2021), one study used β‐Ionone (Dong et al. 2013), one study used crocetin (Oliveira et al. 2017), one study used deinoxanthin (Choi et al. 2014), one study used fucoxanthinol (Terasaki et al. 2017), one study used torulene (Du et al. 2017), one study used torularhodin (Du et al. 2015), and one study used zeaxanthin (Sheng et al. 2020) (Figure 2a). On the other hand, two studies (Loganathan et al. 2015; Moccia et al. 2020) used total mixed carotenoids, Zhao, Gu, et al. (2014a) used β‐carotene, bixin, and astaxanthin, while Zeng et al. (2018) used fucoxanthin and fucoxanthinol as interventions.

Thirteen studies tested the anticancer effects of carotenoids on human breast cancer cell lines (Bakshi et al. 2016; Erden 2020; Gong et al. 2018; Karimian et al. 2022; Kim et al. 2020; Li et al. 2018; Loganathan et al. 2015; Petchsak and Sripanidkulchai 2015; Takeshima et al. 2014; Wang et al. 2022; Wu et al. 2021; Xu et al. 2022), while eight studies tested on gastric cancer cell lines (Dong et al. 2013; Gao et al. 2019; Kim et al. 2021; Park et al. 2019; Sheng et al. 2020; Yu et al. 2018; Zhou et al. 2016, 2019); seven studies used lung cancer cell lines (Fang et al. 2022; Ko et al. 2016; Liao et al. 2016; Luan et al. 2022; Mei et al. 2017; Ming et al. 2021; Yamagata et al. 2017); five studies used liver cancer cell lines (Kavalappa et al. 2019; Kim and Park 2018; Li et al. 2015; Liu et al. 2013; Zeng et al. 2018); four used colon cancer cell lines (Cilla et al. 2015; Hormozi et al. 2019; Manmuan and Manmuan 2019; Terasaki et al. 2017); three studies used blood cancer cell lines (Kim et al. 2017; Moccia et al. 2020; Zhao, Gu, et al. 2014); three studies used cervical cancer cell lines (Gansukh, Mya, et al. 2019; Gansukh, Nile, et al. 2019; Ye et al. 2014); three studies used prostate cancer cell lines (Du et al. 2017; Du et al. 2015; Sun, Zhao, et al. 2020); three studies used glioma cell lines (Liu et al. 2016; Siangcham et al. 2020; Wu et al. 2019); two studies used ovarian cancer cell lines (Su et al. 2019; Xu et al. 2019); two studies used pancreatic cancer cell lines (Bakshi et al. 2020; Jeong et al. 2019); two studies used thyroid cancer cell lines (Tang et al. 2022; Zhang et al. 2016); one study used oral cancer cell line (Wang et al. 2020); one study used bladder cancer cell line (Wang, Zeng, et al. 2014a); one study used skin cancer cell line (Bi et al. 2021) and one study used esophageal cancer cell line (Zhang et al. 2016) (Figure 2b). In contrast, four studies used multiple cancer cell lines, in which Choi et al. (2014) used liver, prostate, and colon cancer cell lines; Lee et al. (2020) used leukemia and colon cancer cell lines; Oliveira et al. (2017) used breast, colon, and stomach cancer cell lines; and lastly, Sawant et al. (2019) used cervical and breast cancer cell lines.

The shortlisted proteins from these 63 studies were first classified based on their reproducibility and expression status. Among the 135 proteins, 22 were RUPs, while 45 proteins were RDPs (Figure 2c; Table S1). Interestingly, there were discrepancies in the expression of three proteins, namely PPARγ, SOD2, and Cyclin D1 (Figure 2c). On the other hand, 71 proteins were only reported in one research paper and were not included in subsequent analyses (Figure 2c; Table S1).

### 3.3 Protein–Protein Interaction Network Analysis of Up‐and Up‐Regulated Proteins

**FIGURE 3:**
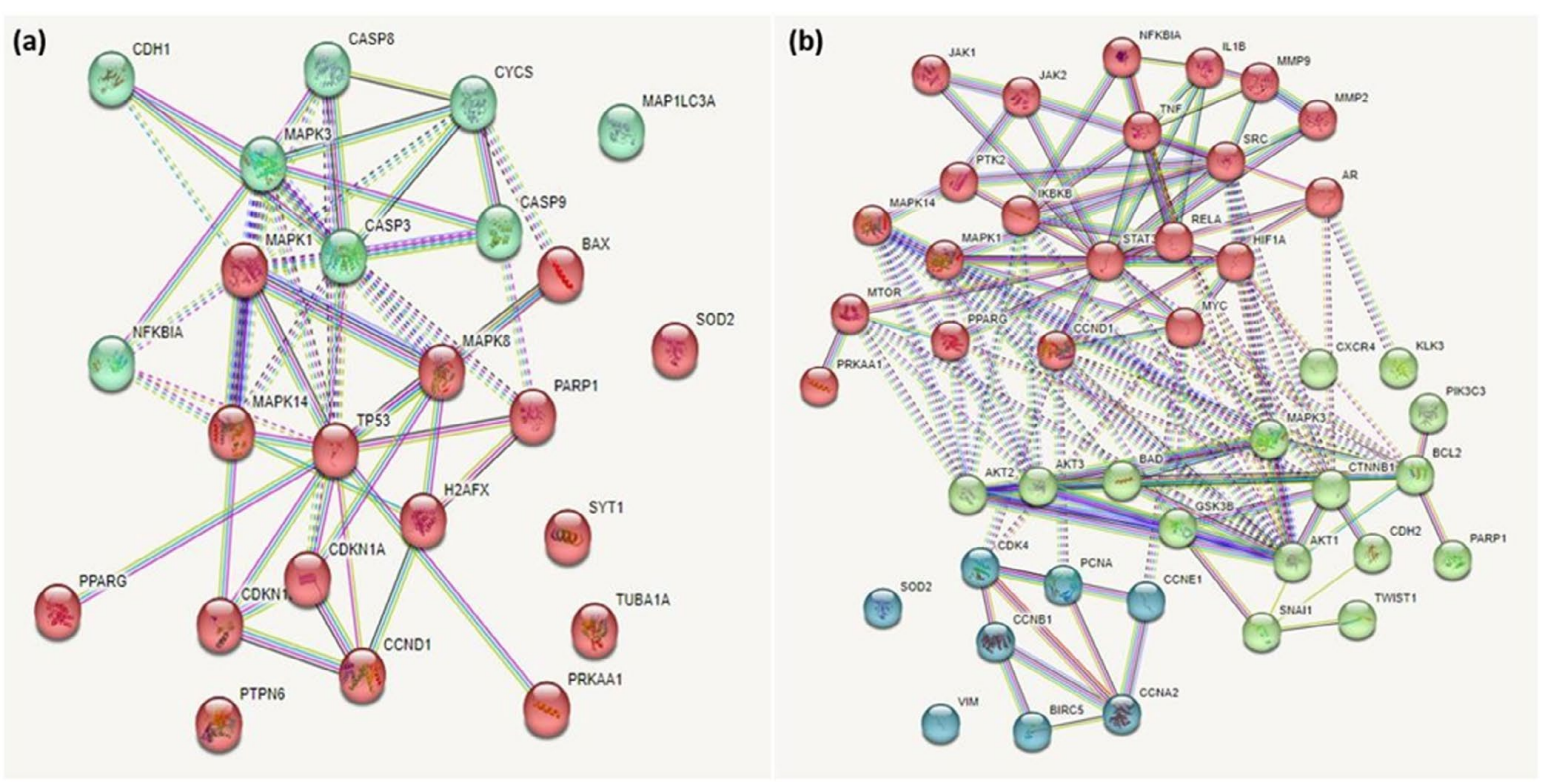
The protein–protein interaction (PPI) networks of reproducible and differentially expressed proteins (RDEPs) were generated via the STRING database. The PPI networks of the (a) reproducible and up‐regulated proteins (RUPs) with two distinct clusters and (b) reproducible and down‐regulated proteins (RDPs) with three distinct clusters. Among the RUP proteins, the red module represents proteins involved in the FoxO signaling pathway, while the green module represents proteins involved in apoptosis. Among the RDP proteins, the red module represents proteins involved in the TNF signaling pathway, the green module represents proteins involved in the PI3k/Akt signaling pathway, and the turquoise module represents proteins involved in cell cycle regulation. All modules were populated using the k‐mean clustering option and the highest confidence score (0.9) in the STRING database. Each node represents a protein with its corresponding gene ID labeled. Filled nodes indicate proteins with known/predicted 3D structures. The edges between nodes represent their respective interactions, whereas the edges indicate known or predicted interactions.

To elucidate the potential anticancer pathway regulated by carotenoids, the RUPs and RDPs were identified from the Draw Venn Diagram *website* (Table S1). The protein–protein interaction (PPI) networks of these RUPs and RDPs were generated with a high confidence value of 0.9 using the STRING database. The PPI network of the RUPs displayed 24 nodes, with an average node degree of 3.33, an average local clustering coefficient of 0.556, and a PPI enrichment p‐value of *p* < 1.0e −16 (Figure 3). On the other hand, the PPI network of RDPs displayed 44 nodes, with an average node degree of 24.6, an average local clustering coefficient of 0.797, and a PPI enrichment p‐value of *p* < 1.0e −16 (Figure 3). Then, the clusters were populated using the k‐means clustering options. Two clusters, namely the Forkhead box transcription factors (FoxO) signaling cluster and apoptosis cluster, were identified in the RUP group (Figure 3a). On the other hand, three clusters, namely the cell cycle cluster, tumor necrosis factor (TNF) signaling cluster, and phosphatidylinositol 3‐kinase/protein kinase B (PI3k/Akt) cluster, were identified in the RDP group (Figure 3b).

Subsequent analysis using the KEGG pathway mapper revealed that the up‐regulated proteins may function in several pathways such as (i) the FoxO signaling pathway (p‐AMPK, JNK, p‐p38, p21, p27, MnSOD) (Figure S1) and (ii) the apoptosis pathway (CASP3, CASP8, CASP9, CyC and IκBα) (Figure S2). On the other hand, the downregulated proteins may function in (i) cell cycle regulation (PCNA, CycE, CycA and CycB) (Figure S3), (ii) the TNF signaling pathway (TNF, NF‐κβ, p38, p‐ERK1/2, p‐IκBα, IKKβ, IL1b, MMP9, TNF, NFκβia) (Figure S4) and (iii) the PI3k/Akt signaling pathway (Akt, GSK3, Myc, AMPK and BAD) (Figure S5).

## 4 Discussion

Carotenoids are naturally occurring lipophilic compounds with strong antioxidant properties (Milani et al. 2017). In recent years, the potential anti‐cancer effects of carotenoids have been extensively studied. However, a comprehensive review of the anti‐cancer pathways modulated by carotenoids is yet to be elucidated. Nonetheless, the advances in pharmacokinetics/pharmacodynamics (PK/PD), such as the establishment of mathematical models to predict in vivo drug efficacy, have greatly improved the physiological relevance of in vitro nutraceuticals data (Alminger et al. 2012; Bouhaddou et al. 2020; De Buck and Mackie 2007; Wienkers and Heath 2005). Therefore, this scoping review identified the proteins and anti‐cancer pathways modulated by carotenoids in cancer cell lines. The scoping review identified 17 carotenoids used in 63 studies, with fucoxanthin, astaxanthin, and crocin being the most commonly used carotenoids (Figure 2a). At the same time, the most common human cancer cell lines investigated were breast, gastric, and lung cancer (Figure 2b). A total of 64 proteins that were reported in two or more research articles were included in the analysis, of which 19 proteins were up‐regulated (RUDPs) while 42 proteins were downregulated (RDPs) in human cancer cells treated with carotenoids (Figure 2c). The expression of three proteins (PPARγ, SOD2 and Cyclin D1) was inconclusive.

As it is widely accepted that the downregulation of Cyclin D1 induces cell cycle arrest, the conflicts in Cyclin D1 expression, as reported by Terasaki et al. (2017), may be due to fucoxanthinol‐induced anoikis. This is not unexpected as there is evidence that suggests the overexpression of cyclin D1 prevents anoikis via inhibition of the transcriptional activity of FoxO proteins [86], which may be a plausible explanation for the upregulation of cyclin D1 observed in the fucoxanthin‐treated colorectal cancer cells. On the other hand, the increase in SOD2 expression observed by Kim et al. (2020), which contradicted findings by Sowmya Sowmya Shree et al. (2017), may potentially be a response to the activation of FoxO proteins rather than be an instigator for the induction of programmed cell death. In contrast, several studies have reported on the dynamic regulation of PPARγ in suppressing the proliferation of cancer cells. PPARγ was found to disrupt the binding of E2F/DP heterodimers to their target genes (Altiok et al. 1997), prevent the phosphorylation of retinoblastoma (Rb) (Wakino et al. 2000) and increase the expression of cyclin‐dependent kinase (CDK) inhibitors (Koga et al. 2001; Motomura et al. 2000). Therefore, the upregulation of PPARγ may enhance the effects on these cell cycle‐associated genes, thereby inducing cell cycle arrest. However, as PPARγ is reported to positively regulate the polarization of the M2 macrophages (Gionfriddo et al. 2020), downregulation of this protein may inhibit the polarization of the M2 macrophages, which may inhibit the progression of cancer in vivo (Na et al. 2013; Siveen and Kuttan 2009; Zhao, Zhang, et al. 2014b). Nonetheless, the conditions under which carotenoids will upregulate or downregulate PPARγ remain unknown.

In the present scoping review, FoxO signaling and apoptosis were the main pathways identified in the RUP group, while cell cycle regulation, PI3k/Akt signaling, and TNF signaling were the main pathways identified in the RDP group (Figure 3). Carotenoid‐induced apoptosis, PI3k/Akt, and TNF signaling have been reviewed elsewhere (Koklesová et al. 2020; Palozza et al. 2006; Zarneshan et al. 2020) and therefore will not be further discussed here. Interestingly, our study revealed that FoxO signaling upregulated p21 and p27 expression (Figure 3A; Figure A1). As cyclin E/CDK2 and cyclin A/CDK2 complexes can be inhibited by p21 and p27 (Toyoshima and Hunter 1994), which in turn are up‐regulated in the presence of nuclear FoxO proteins (Figure A1), our study suggests that cell cycle arrest may be triggered via the activation of FoxO signaling induced by carotenoids.

**FIGURE 4:**
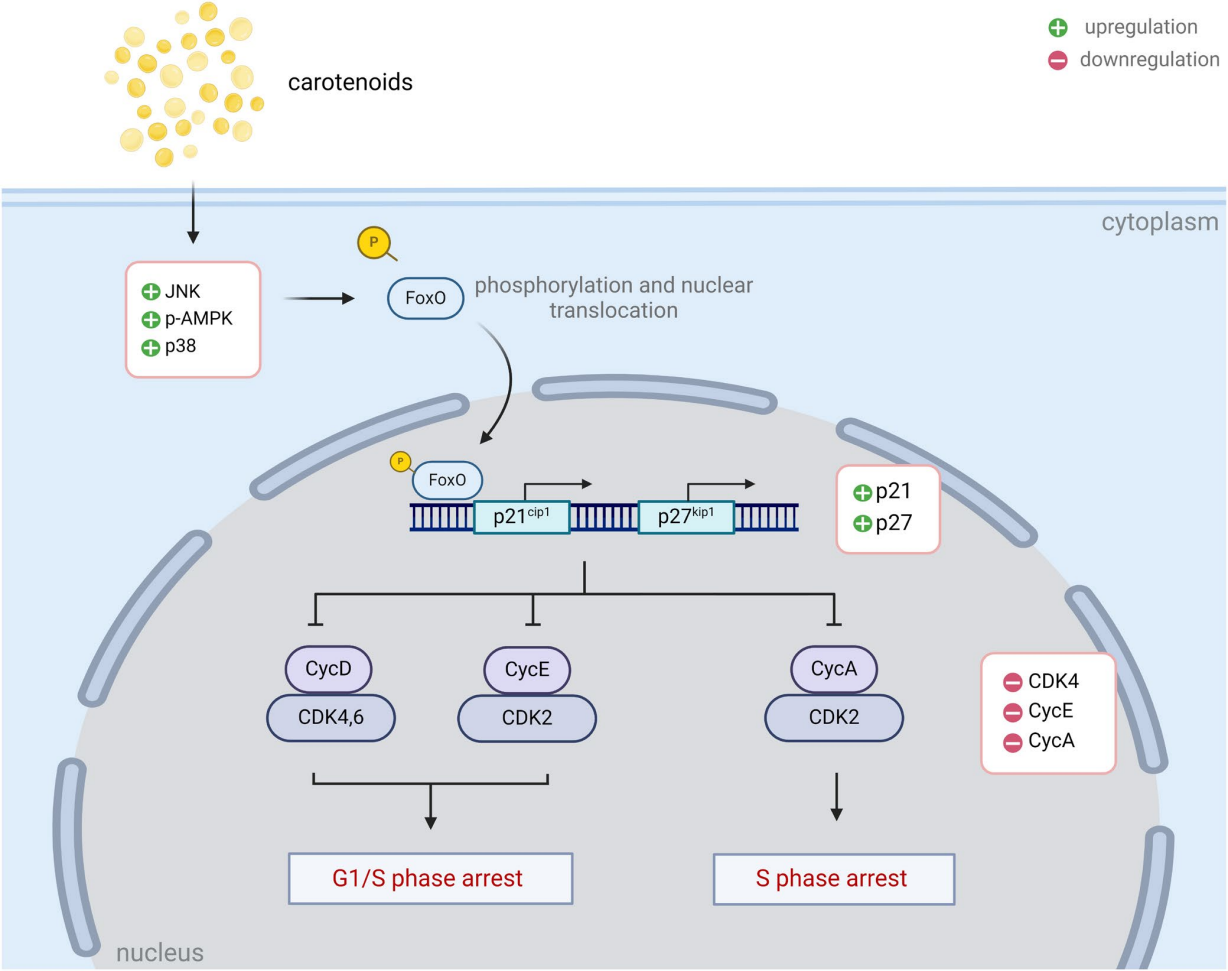
Proposed anticancer mechanism of carotenoids involving FoxO‐mediated cell cycle arrest.

The FoxO signaling pathway consists of the FoxO family, which plays a significant role in the regulation of transcription factors (Farhan et al. 2017). The involvement of FoxO signaling in promoting the anti‐cancer effects of therapeutic agents often begins with the activation of their upstream regulators, namely the AMPK and JNK proteins (Farhan et al. 2017; Wang et al. 2014b), which were also noted in our findings (Figure 3a; Figure A1). The AMPK and JNK proteins are reported to activate FoxO proteins via phosphorylation, leading to the translocation of FoxO proteins into the nucleus (Farhan et al. 2017; Wang et al. 2012). The nuclear FoxO proteins then act as transcription factors that bind to the promoters of various negative cell cycle regulators such as p21 and p27 (Du et al. 2016; Medema et al. 2000), thereby increasing their expression at the transcriptional level and subsequently leading to cell cycle arrest. Noteworthily, the FoxO‐induced cell cycle arrest activated by carotenoids is not limited to the p27‐dependent pathway. For instance, the expression of FoxO4 protein induced cell cycle arrest in p27‐deficient cells by inhibiting cyclin D2 (Schmidt et al. 2002), demonstrating the physiological relevance of carotenoids‐activated, FoxO‐induced cell cycle arrest in the treatment of cancer. Similarly, the ability of carotenoids to activate p21, a well‐known p53 downstream effector, via FoxO also suggests their anti‐tumor potential against p53‐mutant cancer cells. Indeed, the cell lines included in this review consist of different p53 statuses (data not shown). Together with numerous studies (Matsuda et al. 2017; Tinkum et al. 2013), it is suggested that the carotenoids‐modulated FoxO signaling induces cell cycle arrest in cancers by targeting multiple downstream cell cycle‐related proteins (Figure 4).

Cell cycle regulation is a highly coordinated biological process that ensures the sequential progression of cells (Kastan and Bartek 2004). A large number of carotenoids, such as crocin, fucoxanthin, cryptoxanthin, zeaxanthin, capsanthin, and astaxanthin, are capable of downregulating proteins associated with cell cycle regulation. To this end, the regulation of the cell cycle involves the cyclin/CDK complexes, which mediate the transition of the cell cycle from one phase to another via the phosphorylation of target genes. For instance, both the cyclin D1/CDK4 complex and cyclin E/CDK2 phosphorylate the Rb, preventing negative regulation of Rb on the E2F transcription factor, enabling the transition from G1 phase to S phase (Montalto and De Amicis 2020). Similarly, the cyclin A/CDK2 complex phosphorylates the nuclear cdc6, allowing the localisation of cdc6 to the cytoplasm, enabling the progression from the S phase to the G2 phase (Petersen et al. 1999). As such, overexpression of the cyclin/CDK complex may result in uncontrolled growth due to the absence of cell cycle checkpoints. Indeed, numerous studies have documented the impact of overexpression of these cell cycle genes in various cancers such as non‐small‐cell lung cancer (Soria et al. 2000), breast cancer (Keyomarsi and Pardee 1993) and colorectal cancer (Wang et al. 1997). Conversely, the downregulation of these proteins, as observed in this scoping review (Figure 3b; Figure A3), strongly supports the anticancer effects of carotenoids, which appear to involve cell cycle arrest via the downregulation of cell cycle‐promoting factors.

Although FoxO‐mediated programmed cell death has long been established, the relationship between carotenoids and FoxO‐mediated programmed cell death remains elusive. Current evidence on the activation of FoxO by carotenoids mainly centers on oxidative stress regulation and aging (Guvatova et al. 2020; Lashmanova et al. 2015; Yang et al. 2019). Similarly, the tripartite interaction among carotenoids, FoxO signaling, and anticancer effects largely focuses on apoptosis as the resultant biological outcome (Ahmed et al. 2022; Nasimian et al. 2020). Therefore, to the best of our knowledge, this review is the first to propose the activation of FoxO‐mediated cell cycle arrest by carotenoids.

Due to the highly heterogeneous nature of cancer and the distinct pharmacological properties of the tested compounds, the upstream signaling involved in the activation of FoxO‐mediated cell cycle arrest varies widely in current literature. Dilmac et al. (2022) revealed that the activation of FOXO proteins by SIRT1 inhibition activated p53 alongside p21 in in vivo models of breast cancer. Moreover, the same study also observed decreased mRNA expressions of the FoxO proteins in triple‐negative breast cancer (TNBC) patients (Dilmac et al. 2022), highlighting their tumor suppressor role in breast cancer. On the other hand, Zhao et al. (2016) identified miR‐411 as the key regulator of FoxO1 protein in lung cancer. Interestingly, mounting evidence has suggested that the suppression of Akt activated FoxO via nuclear translocation in breast cancer (Hill et al. 2014; Scodelaro Bilbao and Boland 2013), gastric cancer (Du et al. 2022; Lin et al. 2019) and lung cancer (Liu et al. 2012; Maekawa et al. 2009; Yu et al. 2017). Moreover, FoxO proteins also resulted in increased sensitization of non‐small‐cell lung cancer (NSCLC) cells to cisplatin (Sun, Zhang, et al. 2020). Therefore, the results of our study (Figure A1; Figure A5) agree with current findings indicating that the PI3K/Akt pathway acts as a key negative regulator of FoxO signaling in cancer cells. Taken together, the PI3K/Akt/FoxO axis in cell cycle regulation may be a promising target for drug development due to its involvement in multiple anticancer mechanisms in cancer cells.

Although FoxO‐specific anticancer agents are lacking, FoxO mediates the effects of several anticancer compounds, such as genotoxic agents, namely doxorubicin (Rosaline C.‐Y. Hui et al. 2008), PI3k/Akt inhibitors namely BEZ235 (Lin et al. 2014), and tyrosine kinase inhibitors, namely imatinib (Wei et al. 2013). In particular, due to its central role in cancer pathway activation, clinical evaluation with various PI3k/Akt inhibitors is currently ongoing. For instance, alpelisib (BYL719) is an oral PI3K/Akt inhibitor that targets the alpha isoform of PI3K (p100α). Phase 3 clinical trials have demonstrated the efficacy of alpelisib against hormone receptor (HR)‐positive, human epithelial growth factor 2 (HER2)‐negative breast cancer {André, 2019 #2846}, leading to its approval by the FDA in 2019 {Alves, 2023 #2847}. Similarly, preclinical combination studies of alpelisib and cetuximab inhibited EGFR phosphorylation, resulting in inhibition of mTOR activation and subsequent cell death in squamous cell carcinoma cells and tumors (Elkabets et al. 2015). On the other hand, large‐scale clinical trials have demonstrated that cell cycle inhibitors, such as the FDA‐approved abemaciclib and ribociclib, which are CDK4/6 inhibitors, significantly extended progression‐free survival of breast cancer patients (Hortobagyi et al. 2016; Sledge et al. 2017). The successful clinical trials of PI3k/Akt inhibitors and cell cycle inhibitors in breast cancer management strongly support our suggestion that targeting the PI3K/Akt/FoxO axis can induce cell cycle arrest, highlighting its translational potential in cancer treatment. Nonetheless, although the dual treatment of a PI3K/Akt inhibitor and a cell cycle inhibitor demonstrated synergistic effects against preclinical breast cancer and head and neck squamous cell carcinoma {Remer, 2020 #2848;Teo, 2017 #2849}, clinical use of this combination has resulted in unexpected toxicity in breast cancer patients {Tolaney, 2021 #2850}. While spontaneous or acquired resistance may explain the observation {Elkabets, 2015 #2845}, the exact mechanism responsible for the toxicity is yet to be determined. Moreover, the clinical evidence on the efficacy of these inhibitors is largely based on breast cancer patients. Thus, their potential efficacy in other cancer types remains unknown. Similarly, whether they rely on the same mechanism of action in a different tumor microenvironment remains uncertain.

To our best knowledge, this is the first review to propose the role of carotenoids in activating FoxO‐induced cell cycle arrest in cancer cells. However, our findings should be interpreted with careful consideration due to several limitations. Due to the high heterogeneity of cancer, including all types of carotenoids and cancer in our search methodology might have excluded several carotenoid‐ or cancer‐specific pathways. This, however, can also be interpreted as a strength as it allowed us to identify common pathways that are shared across different types of carotenoids and cancers. Furthermore, this review did not include in vivo cancer models, which could potentially produce a different outcome when considering their physiological relevance. This review also did not restrict the origin of carotenoids (plant, microorganism, synthetic) which could have altered the signaling pathways due to the presence of various stereoisomers from different sources.

Moreover, whether the source of carotenoids influences its anticancer potential remains to be determined. Although carotenoids have demonstrated significant anticancer potential in vitro, past and ongoing clinical trials largely focus on their bioavailability in human subjects (Martini et al. 2022). On the other hand, current commercially available β‐carotenes are typically synthesized through chemical reactions (synthetic β‐carotene) while the production of natural β‐carotene relies on extraction from palm oil (Baharin et al. 2001) or cultivating algae or genetically engineered yeasts (Pouzet et al. 2022; Raja et al. 2007). However, despite being identical in chemical formula, both forms of β‐carotene display different physiochemical properties and bioavailability, as documented by Ben‐Amotz et al. (1989) and Capelli et al. (2013). Such distinct results can be attributed to the stereochemistry of the different forms of β‐carotene, where synthetic β‐carotene consists of only all‐cis configuration while natural β‐carotene consists of all‐trans, 9‐cis, and 13‐cis configurations (Honda 2021; Patrick 2000). As such, the proportion of isomers may be different when ingested (Ben‐Amotz et al. 1989; Stahl et al. 1992). Nonetheless, the extent to which each isomer contributes to the anticancer effect and the source of carotenoids used in these clinical trials remains unclear.

Furthermore, clinical evidence on the role of carotenoids in cancer prevention and treatment remains divided. The North Carolina‐Louisiana prostate cancer project (PCaP) observed the protective effect of dietary lycopene and β‐cryptoxanthin against aggressive prostate cancer among African‐American and European‐American men (Antwi et al. 2016). Similarly, a meta‐analysis published as part of the World Cancer Research Fund/American Institute for Cancer Research (WCRF/AICR) Continuous Update Project (CUP) suggested an inverse dose–response relationship between blood carotenoids and lung cancer risk (Abar et al. 2016). In contrast, the Isotretinoin‐Basal Cell Carcinoma Prevention Trial found no association between serum carotenoid level and basal or squamous cell carcinoma (Dorgan et al. 2004). Consistent with a previous study, long‐term β‐carotene supplementation yielded no effect on liver cancer incidence (Lai et al. 2014). However, all authors noted inconclusive findings due to the lack of strong mechanistic evidence (Abar et al. 2016; Antwi et al. 2016; Dorgan et al. 2004; Lai et al. 2014). Indeed, only the ProDiet randomized controlled trial conducted in recent years suggested that the reduced prostate cancer risk associated with increased lycopene intake may be due to decreased pyruvate levels by lycopene (Beynon et al. 2019). Coincidentally, an ex vivo study observed differential regulation of cell cycle‐ and apoptotic‐related genes by sera lycopene in prostate cancer cells (Talvas et al. 2010). Nonetheless, the complexity in measuring the bioavailability of carotenoids (Faulks and Southon 2005), coupled with the highly heterogeneous populations and methodologies adopted by different studies, necessitates more refined studies to determine the association between carotenoids and cancer as well as their anticancer potential in the clinical setting.

## 5 Conclusion

Although the use of carotenoids as a potential anticancer agent has been underway, a comprehensive review of their anticancer mechanisms remains unknown. The present scoping review identified 64 replicable and differentially expressed proteins across 63 studies. Subsequent analyses revealed that FoxO signaling and apoptosis were among the upregulation clusters responsible for the anti‐cancer mechanisms of carotenoids, while TNF signaling, PI3k/Akt signaling, and cell cycle regulation were among the downregulation clusters responsible for the anti‐cancer mechanisms of carotenoids. Noteworthily, carotenoids may induce cell cycle arrest by activating the FoxO signaling pathway. Thus, utilizing carotenoids and targeting the FoxO signaling pathway may be a potential strategy for the treatment of cancer patients. In the era of integrative healthcare, carotenoids may serve as adjuncts to conventional treatment strategies as part of a holistic approach to combat cancer. In addition to the promising anticancer effects, their distinct targeting mechanism, synergistic effects, and wide commercial availability may significantly improve clinical outcomes and minimize recurrence. However, further studies are required for the validation of FoxO‐induced cell cycle arrest by carotenoids. Similarly, clinical data on the mechanisms, bioavailability, optimal dosage, and long‐term safety profile of carotenoids are also required to ensure a successful bench‐to‐bedside journey.

## Author Contributions

**Zi Xin Lee:** data curation (equal), formal analysis (equal), investigation (equal), validation (equal), writing – original draft (lead). **Hanting Guo:** data curation (equal), investigation (equal), writing – review and editing (equal). **Aaron Deming Looi:** methodology (equal), resources (equal), software (equal), writing – review and editing (equal). **Saatheeyavaane Bhuvanendran:** methodology (equal), project administration (equal), supervision (equal), writing – review and editing (equal). **Kasthuri Bai Magalingam:** resources (equal), software (equal), supervision (equal), writing – review and editing (equal). **Wai Leng Lee:** supervision (equal), writing – review and editing (equal). **Ammu Kutty Radhakrishnan:** conceptualization (equal), data curation (equal), funding acquisition (equal), project administration (equal), supervision (equal), writing – review and editing (equal).

## Conflicts of Interest

The authors declare no conflicts of interest.

## Supporting information


**Figure S1.** KEGG Mapper analysis of identified proteins (highlighted in pink) involved in the FoxO signaling pathway.

**Figure S2.** KEGG Mapper analysis of identified proteins (highlighted in pink) involved in the apoptosis pathway.

**Figure S3.** KEGG Mapper analysis of identified proteins (highlighted in pink) involved in the cell cycle pathway.

**Figure S4.** KEGG Mapper analysis of identified proteins (highlighted in pink) involved in the TNF signaling pathway.

**Figure S5.** KEGG Mapper analysis of identified proteins (highlighted in pink) involved in the PI3k/Akt signaling pathway.

**Table S1.** List of gene IDs and their corresponding reproducibility and expression status.

## Data Availability

These data were derived from the following resources available in the public domain (Table 2).
