# Supplementary figures and images for: Carotenoids Modulate FoxO‐Induced Cell Cycle Arrest in Human Cancer Cell Lines: A Scoping Review
Source: Food Sci Nutr. 2025 Mar 28;13(4):e70100. doi: 10.1002/fsn3.70100 (PMC11953061; doi:10.1002/fsn3.70100)

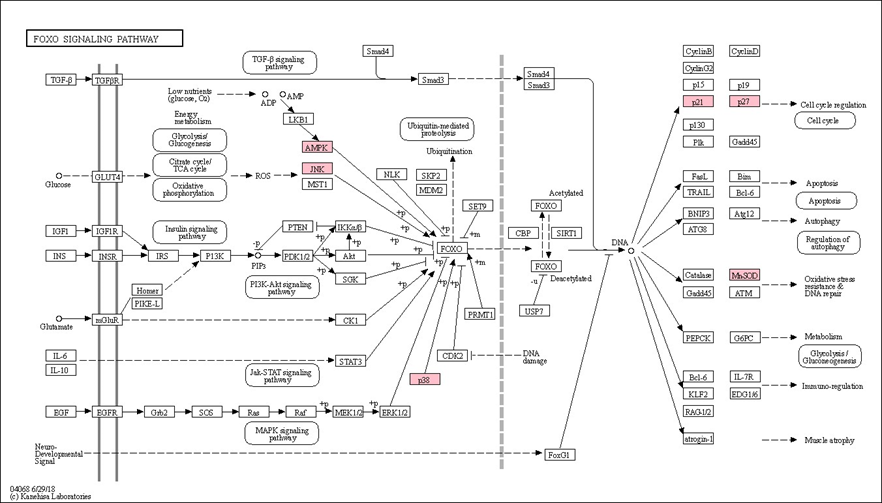

Supplement: Supplementary file 1 — Figure S1. KEGG Mapper analysis of identified proteins (highlighted in pink) involved in the FoxO signaling pathway. [file FSN3-13-e70100-s001.png]

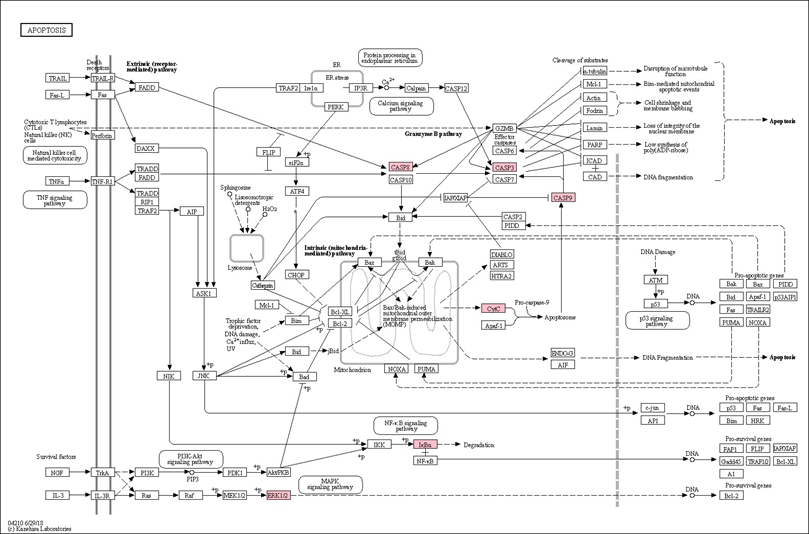

Supplement: Supplementary file 2 — Figure S2. KEGG Mapper analysis of identified proteins (highlighted in pink) involved in the apoptosis pathway. [file FSN3-13-e70100-s005.png]

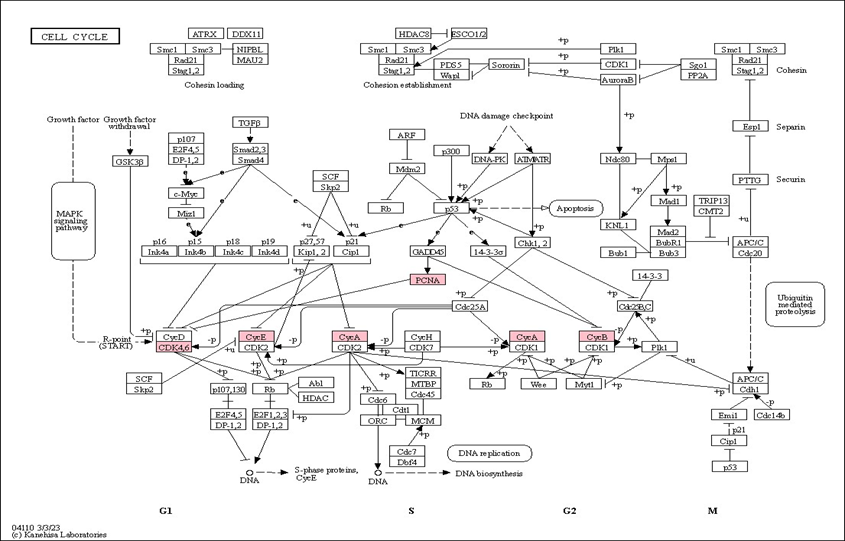

Supplement: Supplementary file 3 — Figure S3. KEGG Mapper analysis of identified proteins (highlighted in pink) involved in the cell cycle pathway. [file FSN3-13-e70100-s002.png]

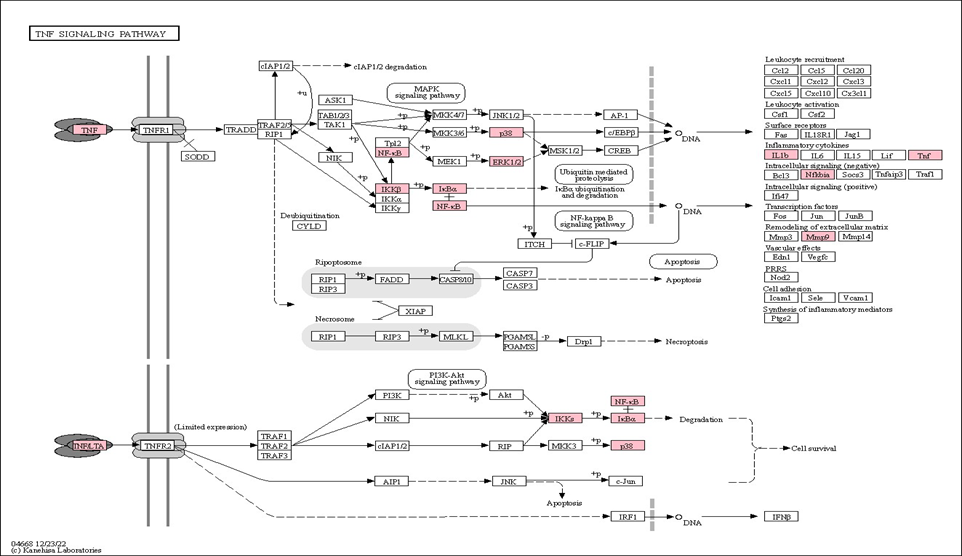

Supplement: Supplementary file 4 — Figure S4. KEGG Mapper analysis of identified proteins (highlighted in pink) involved in the TNF signaling pathway. [file FSN3-13-e70100-s004.png]

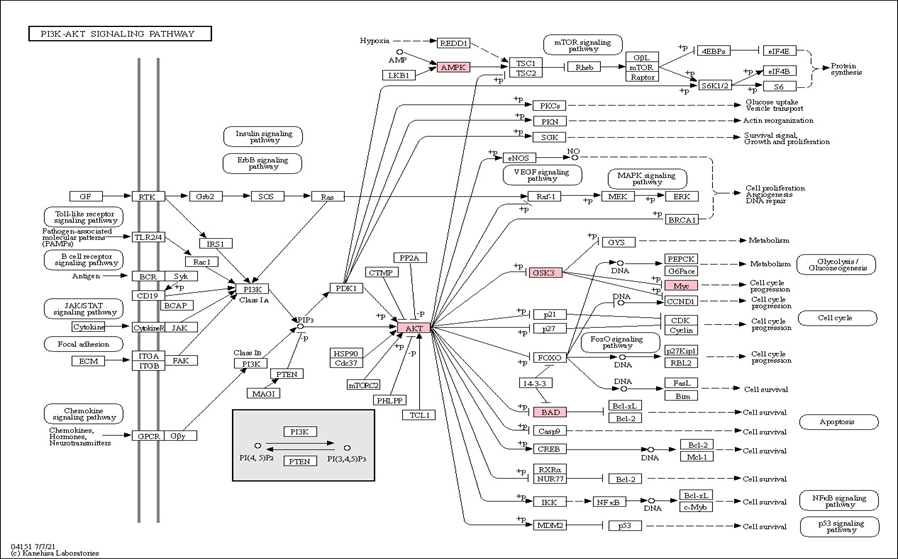

Supplement: Supplementary file 5 — Figure S5. KEGG Mapper analysis of identified proteins (highlighted in pink) involved in the PI3k/Akt signaling pathway. [file FSN3-13-e70100-s006.png]
