# Supplementary material for: Carotenoids Modulate FoxO‐Induced Cell Cycle Arrest in Human Cancer Cell Lines: A Scoping Review
Source: Food Sci Nutr. 2025 Mar 28;13(4):e70100. doi: 10.1002/fsn3.70100 (PMC11953061; doi:10.1002/fsn3.70100)
Supplement: Supplementary file 6 — Table S1. List of gene IDs and their corresponding reproducibility and expression status. [file FSN3-13-e70100-s003.pdf]

Table S1. List of gene IDs and their corresponding reproducibility and expression status.

Description	Human Gene ID
Reproducible**	GSK3B, CCNA2, MMP2, AKT1, TBA1A, PCNA, MYC, IKKB, PARP1, CCNB1, CDN1B, PK3C3, CADH1, VEGFA, ANDR, CASP9, MMP9, CADH2, P53, HIF1A, SRC, SODM, CTNB1, CDN1A, IL1B, PTN6, CYC, PPARG, AKT2, H TWST1, MK03, JAK1, IKBA, MK08, MK01, STAT3, TNFA, CXCR4, MTOR, MK14, TF65, BIRC5, SNAI1, KLK3, VIME, CDK4, AKT3, BAD, H2AX, FAK1, CCND1, CASP3, BAX, MLP3A, JAK2, IKBA (cleaved), AAPK1, BCL2, CCNE1, PARP1 (cleaved), CASP8, MK14 (cleaved),
Non-reproducible*	PROM1, BIRC3, UROK, IKBL1, ATG2B, SODC, RUVB1, ZEB1, GRP75, BUB1B, KLF5, COF1, ATM, TIMP2, BIRC2, MD2L1, NOTC3, PAXI, CD44, MCL1, CASP6, SYT1, FINC, XBP1, SQSTM, CDK6, RECA TYSY, ACTA, KPCT, PUMA GSHB, RIPK3, CH60, EF2K, KS6B1, EZH2, ITB1, TRAF2, MLKL, FADD, SOX2, LDHD, MARE1, CATA, NFKB1, PTN4, IL6, MLP3B, MP2K1, JAG2, GPX3, HMOX1, DLL1, NF2L2, RIPK1, TRAF1, CDK2, CDK1, CAV1, BECN1, ATR, JAG1, C163A, KAPCA, XIAP, HES1, SYT1, MP2K2, CHD5,
Reproducible and up-regulated	TBA1A, AAPK1 (cleaved), BAX, CASP3, CASP8, CASP9, PARP1 (cleaved), CCND1, CYC, CADH1, MK01 (cleaved), MK03 (cleaved), H2AX, IKBA, MK08, MLP3A, CDN1A, CDN1B, MK14, P53, PPARG, PTN6, SODM,
Reproducible and downregulated	AKT1, AKT2, AKT3, AAPK1, ANDR, BCL2, CDK4, PARP1, MYC, CXCR4, CCNA2, CCNB1, CCND1, CCNE1, MK03, MK01, FAK1, GSK3B, HIF1A, IKKB, IL1B, JAK2, MMP2, MMP9, MTOR, CADH2, TF65, BAD, IKBA, SRC, PK3C3, MK14 (cleaved), PPARG, PCNA, KLK3, SNAI1, SODM, STAT3, BIRC5, TNFA, TWST1, VEGFA, VIME, CTNB1, JAK1, IKBA (cleaved),

** identified in two or more papers included in this study

* identified in one paper included in this study
